# Supplementary figures and images for: Viromes of one year old infants reveal the impact of birth mode on microbiome diversity
Source: PeerJ. 2018 May 7;6:e4694. doi: 10.7717/peerj.4694 (PMC5944432; doi:10.7717/peerj.4694)

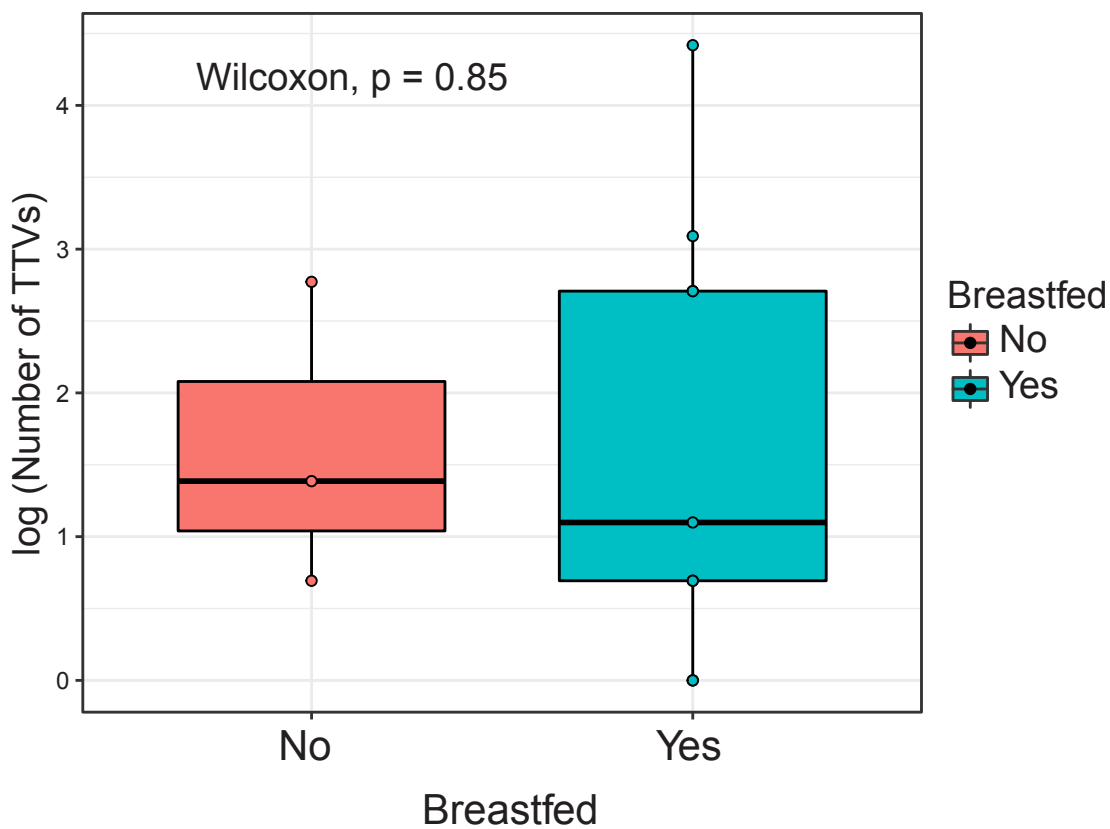

Supplement: Figure S1 — Boxplot of the number of detectable homologues of Torque Teno Virus (TTV) ORF1 in each sample by breastfeeding status. [file peerj-06-4694-s006.pdf]

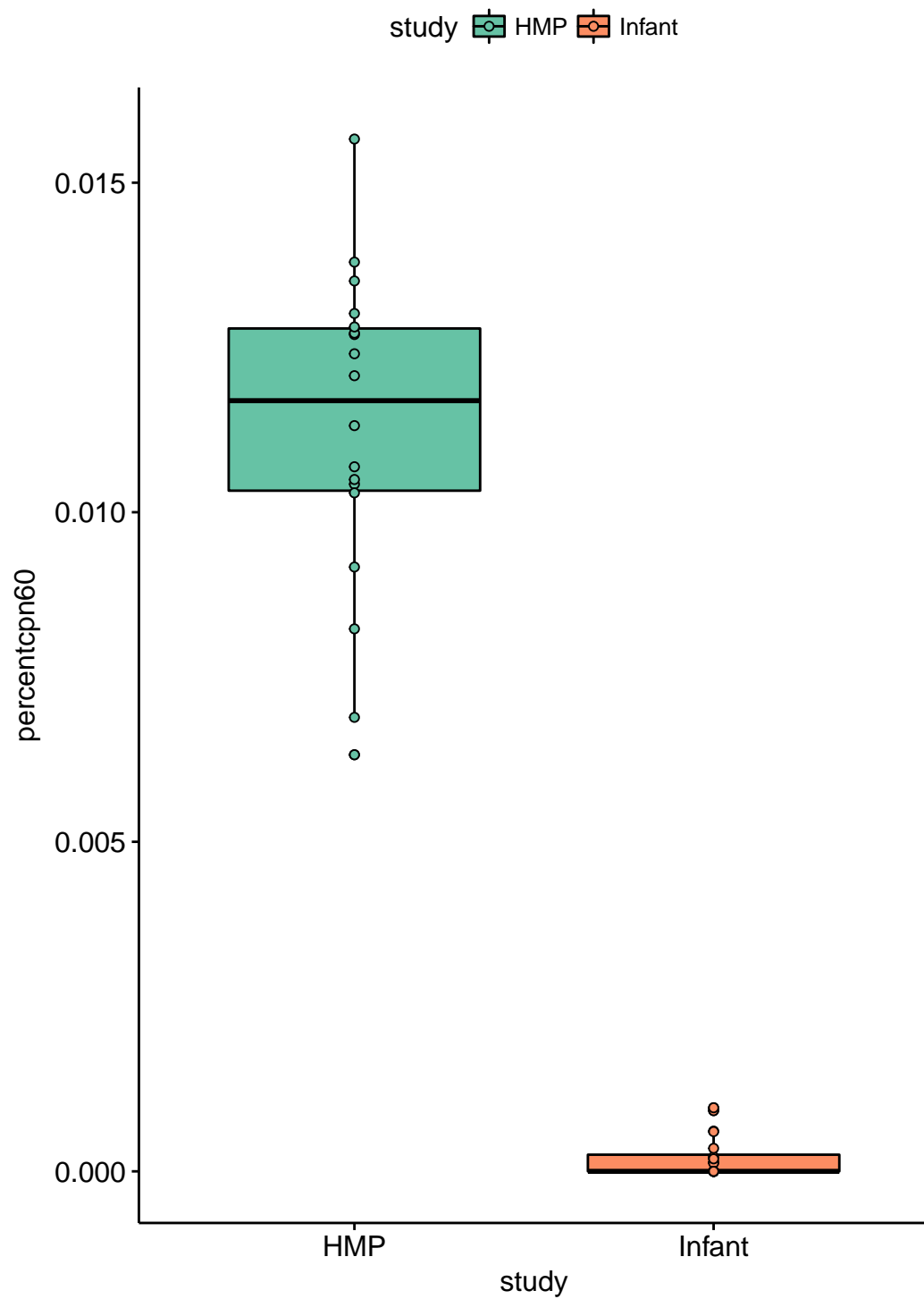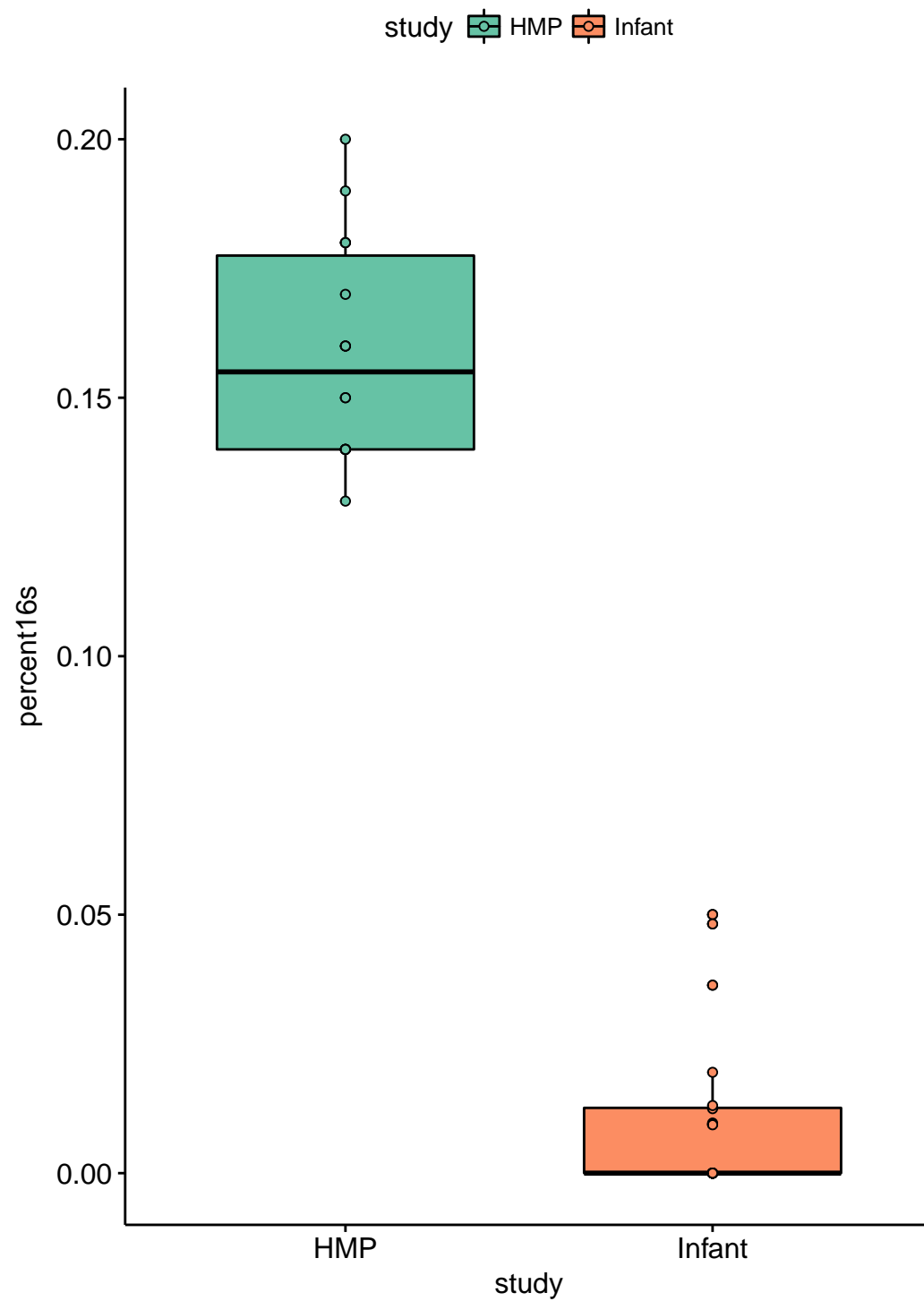

Supplement: Figure S3 — Percentage of 16S rRNA and cpn60 per sample in viromes sequenced in this study, as well as whole shotgun metagenomics samples from the Human Microbiome Project for comparsion. [file peerj-06-4694-s008.pdf]

# DNA Virome

a

Wilcoxon,  $p = 0.00077$

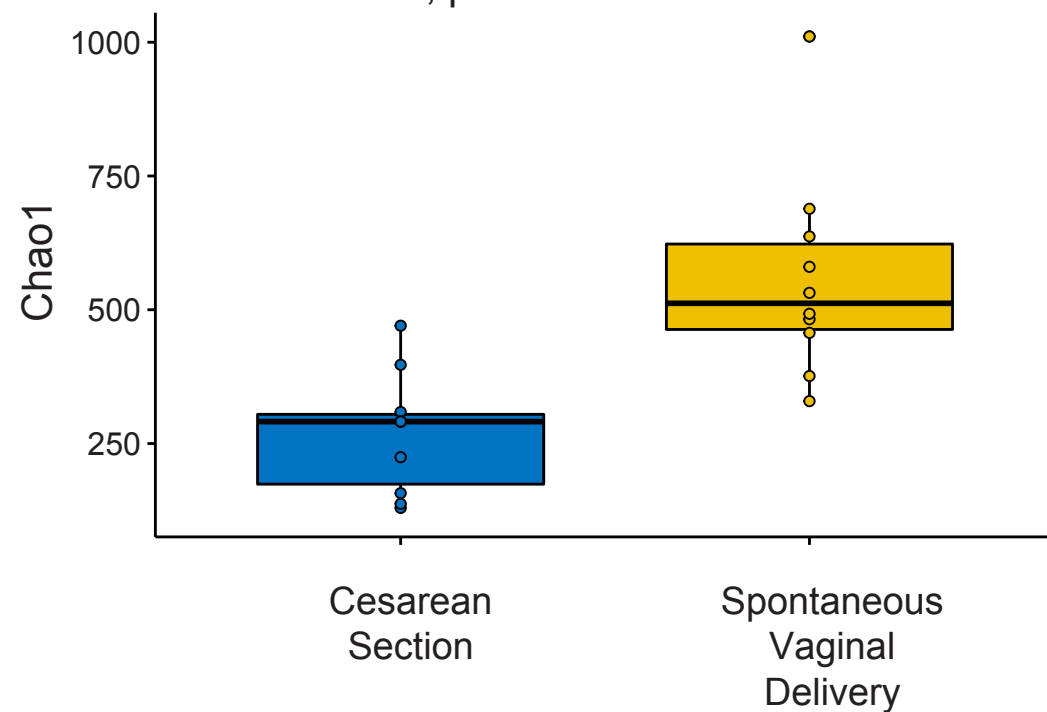

# 16S rRNA

b

Wilcoxon,  $p = 0.89$

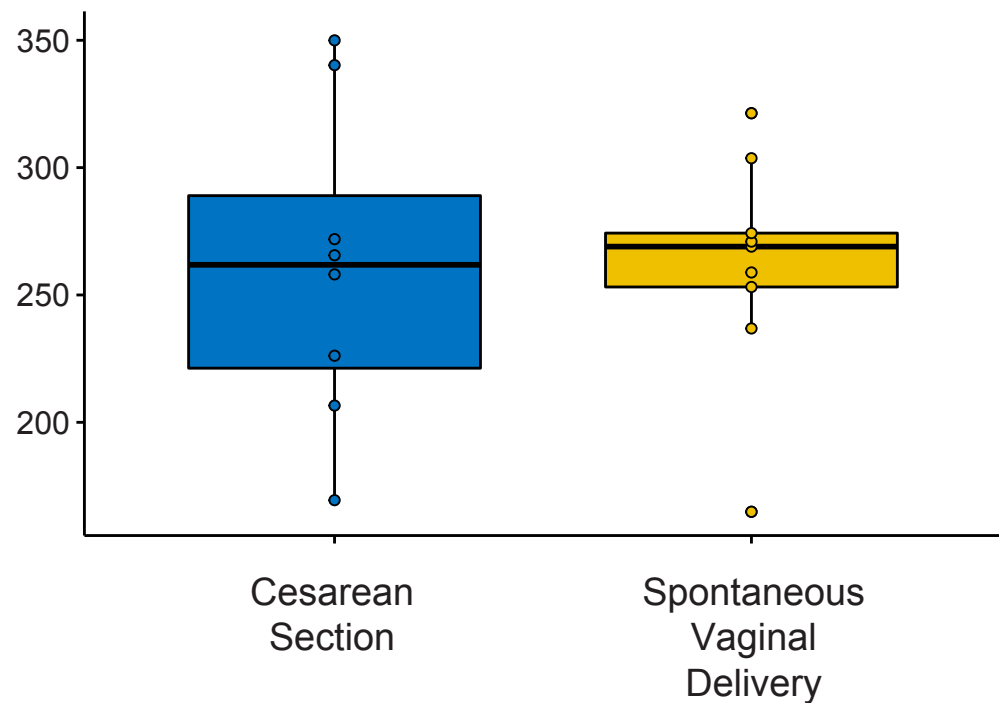

Supplement: Figure S4 — Each point represents the Chao1 diversity estimate based on the assembled sequences per infant faecal sample. [file peerj-06-4694-s009.pdf]
