# Supplementary material for: Viromes of one year old infants reveal the impact of birth mode on microbiome diversity
Source: PeerJ. 2018 May 7;6:e4694. doi: 10.7717/peerj.4694 (PMC5944432; doi:10.7717/peerj.4694)
Supplement: Figure S2 — Heatplot demonstrating the level of nucleotide identity across the CrAssphage genomes assembled in this study, the original CrAssphage and from the Reyes et al., 2015 Malawi infant study. [file peerj-06-4694-s007.pdf]

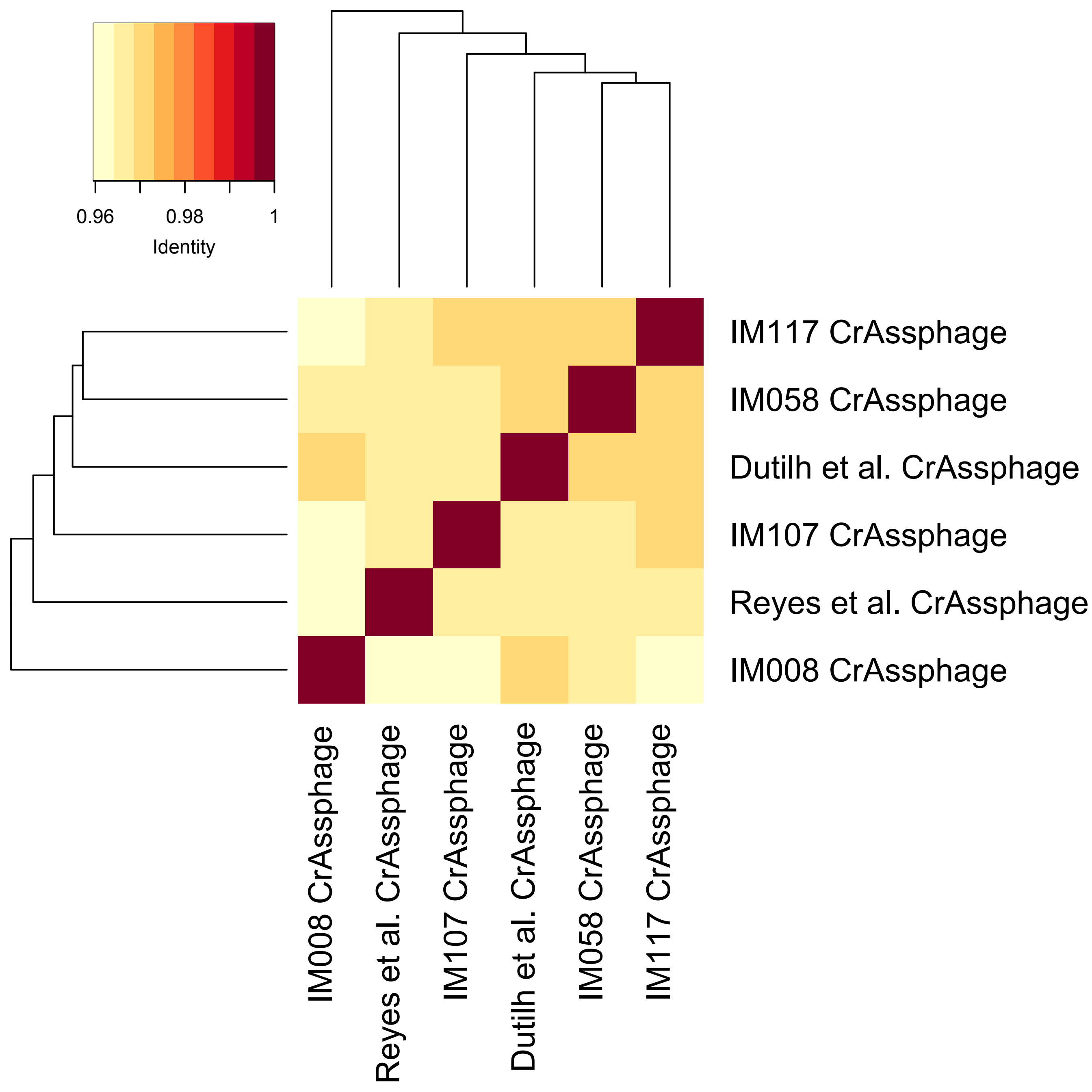
0.96
0.98
1
Identity
IM117 CrAssphage
IM058 CrAssphage
Dutilh et al. CrAssphage
IM107 CrAssphage
Reyes et al. CrAssphage
IM008 CrAssphage
